# Combining Computational Fluid Dynamics, Structural Analysis, and Machine Learning to Predict Cerebrovascular Events: A Mild ML Approach

**DOI:** 10.3390/diagnostics14192204

**Published:** 2024-10-02

**Authors:** Panagiotis K. Siogkas, Dimitrios Pleouras, Vasileios Pezoulas, Vassiliki Kigka, Vassilis Tsakanikas, Evangelos Fotiou, Vassiliki Potsika, George Charalampopoulos, George Galyfos, Fragkiska Sigala, Igor Koncar, Dimitrios I. Fotiadis

**Affiliations:** 1 Unit of Medical Technology and Intelligent Information Systems, Department of Materials Science and Engineering, University of Ioannina, 45110 Ioannina, Greece; psiogkas@uoi.gr (P.K.S.); dipleouras@gmail.com (D.P.); bpezoulas@gmail.com (V.P.); kigkavaso@gmail.com (V.K.); vasilistsakanikas@gmail.com (V.T.); vafotiou@gmail.com (E.F.); vpotsika@gmail.com (V.P.); 2 First Propedeutic Department of Surgery, National and Kapodistrian University of Athens, 11527 Athens, Greece; charalampopoulosg@gmail.com (G.C.); georgegalyfos@hotmail.com (G.G.); drfsigala@yahoo.gr (F.S.); 3 Department of Vascular and Endovascular Surgery, Faculty of Medicine, University of Belgrade, 11000 Belgrade, Serbia; dr.koncar@gmail.com; 4 Biomedical Research Institute—Foundation for Research and Technology—Hellas, 45110 Ioannina, Greece

**Keywords:** computational fluid dynamics (CFD), machine learning (ML), cerebrovascular events

## Abstract

**Background/Objectives**: Cerebrovascular events, such as strokes, are often preceded by the rupture of atherosclerotic plaques in the carotid arteries. This work introduces a novel approach to predict the occurrence of such events by integrating computational fluid dynamics (CFD), structural analysis, and machine learning (ML) techniques. The objective is to develop a predictive model that combines both imaging and non-imaging data to assess the risk of carotid atherosclerosis and subsequent cerebrovascular events, ultimately improving clinical decision-making. **Methods:** A multidisciplinary approach was employed, utilizing 3D reconstruction techniques and blood-flow simulations to extract key plaque characteristics. These were combined with patient-specific clinical data for risk evaluation. The study involved 134 asymptomatic individuals diagnosed with carotid artery disease. Data imbalance was addressed using two distinct approaches, with the optimal method chosen for training a Gradient Boosting Tree (GBT) classifier. The model’s performance was evaluated in terms of accuracy, sensitivity, specificity, and ROC AUC. **Results:** The best-performing GBT model achieved a balanced accuracy of 88%, with a ROC AUC of 0.92, a sensitivity of 0.88, and a specificity of 0.91. This demonstrates the model’s high predictive power in identifying patients at risk for cerebrovascular events. **Conclusions:** The proposed method effectively combines CFD, structural analysis, and ML to predict cerebrovascular event risk in patients with carotid artery disease. By providing clinicians with a tool for better risk assessment, this approach has the potential to significantly enhance clinical decision-making and patient outcomes.

## 1. Introduction

Carotid atherosclerosis and the rupture of atheromatic plaques represent a complex occurrence influenced by numerous factors, involving both biological components and biomechanical forces acting on the arterial wall. Specific to each patient, factors like arterial shape, peak systolic blood velocity, pressure, plaque makeup, and structure are crucial in determining the mechanical strains experienced by the arterial wall. High wall shear stress (WSS) and increased plaque structural stress (PSS) due to unstable plaque components are recognized as factors contributing to plaque rupture. Moreover, various measures of plaque accumulation, such as volume, cross-sectional area, or thickness of plaque elements, as well as the presence, size, or volume of specific plaque tissues, have been linked to past, present, and recurrent symptoms or events, including stroke.

In the current literature, there are several studies that explore the relationship between plaque characteristics and the risk of plaque rupture, as well as the biomechanical stresses associated with this risk. Takaya et al. [1] conducted a prospective study on asymptomatic patients with carotid stenosis, finding significant associations between baseline MRI-detected plaque features and subsequent cerebrovascular events. Similarly, Zhao et al. [2] identified associations between carotid plaque characteristics and acute cerebral infarct sizes.

Gupta et al. [3] conducted a meta-analysis confirming that certain plaque components, including intraplaque hemorrhage (IPH), lipid-rich necrotic core (LRNC), and thin/ruptured fibrous cap (FC), are predictors of future stroke or transient ischemic attack (TIA). Esposito-Bauer et al. [4] and Selwaness et al. [5] also found associations between plaque composition and cerebrovascular events. Furthermore, studies by Sun J. et al. [6], Sun B. et al. [7], Xia et al. [8], and Cui et al. [9] explored various aspects of plaque characteristics and their associations with cardiovascular outcomes. Notably, IPH, LRNC, and FC rupture were strongly associated with adverse outcomes in these studies.

On the biomechanical side, Cheng et al. [10] and Li et al. [11] investigated stress distributions within plaques, finding higher circumferential stress in ruptured plaques. Groen et al. [12] and Kock et al. [13] used imaging data to simulate longitudinal fibrous cap stresses, identifying potential indicators of plaque vulnerability. Moreover, Tang et al. [14] and Teng et al. [15] employed finite element analysis (FEA) and computational fluid dynamics (CFD) to assess plaque wall stress and flow-induced stresses, revealing associations with plaque rupture. Other studies, including those by Ohayon et al. [16], Huang et al. [17], and Wang et al. [18], further explored stress distributions within plaques using FSI simulations based on imaging data. Furthermore, studies by Tuenter et al. [19], Costopoulos et al. [20], and Doradla et al. [21] investigated associations between wall shear stress (WSS), plaque composition, and plaque vulnerability, providing insights into the biomechanical mechanisms underlying plaque rupture.

Curcio et al. [22] introduced a patient-specific computational approach to assess the vulnerability of carotid artery plaques. By employing geometric modeling and structural simulation techniques, they identified regions of elevated stress within the artery wall, particularly around plaques, indicating potential rupture areas. In plaques with lipid composition and heterogeneity, maximum stresses were concentrated within the fibrous cap. Jansen et al. [23] developed a tissue-engineered model of atherosclerotic plaque caps, incorporating microcalcifications to evaluate their influence on mechanical properties. Their model confirmed the adverse role of microcalcifications in increasing the risk of cap rupture and offers insights into tissue mechanics involving soft tissue calcification. Additionally, Zhang et al. [24] devised a 3D carotid plaque radiomics model using high-resolution magnetic resonance imaging (HRMRI), which outperformed traditional models in identifying vulnerable plaques, demonstrating superior performance in both training and test cohorts. These studies collectively advance the understanding and assessment of carotid artery plaque vulnerability through computational, tissue-engineering, and radiomics approaches, offering valuable insights for enhanced clinical management. Overall, these studies contribute to our understanding of the complex interplay between plaque characteristics, biomechanical stresses, and the risk of plaque rupture, offering valuable insights for risk assessment and management in patients with atherosclerotic disease.

Significant advancements in using machine learning (ML) models for predicting cerebrovascular events such as stroke, transient ischemic attacks (TIA), Amaurosis Fugax, etc. related to carotid artery disease have been witnessed in reviewing current literature. Studies have employed diverse ML techniques, integrating both clinical data and imaging inputs to enhance predictive accuracy. For instance, Wu et al. [25] and Weng et al. [26] utilized clinical and imaging data, demonstrating improved model performance in predicting major adverse cardiovascular and cerebrovascular events. Moreover, research incorporating radiomics [27], underscores the potential of advanced imaging features in refining ML predictions.

Additionally, the integration of explainable ML models [28], provides significant insights into early screening and risk assessment for carotid atherosclerosis. Other notable contributions include hybrid modeling approaches combining clinical and imaging data, enhancing the prediction of stroke risk [29]. Despite the limited focus on simulation-based inputs specifically for carotid artery disease, the existing literature underscores the transformative potential of ML in predicting cerebrovascular events, paving the way for more personalized and precise healthcare interventions.

In this context, we propose a machine learning (ML) model for predicting forthcoming cerebrovascular events which combines as input hemodynamic parameters obtained through finite element analysis with non-imaging characteristics deriving from eCRF recordings and demographic data, a combination that constitutes its uniqueness and novelty compared to other proposed models deriving from current literature. This model provides a detailed understanding of the intricate connection between biomechanical stresses and plaque characteristics, making it an essential tool for assessing the probability of an impending cerebrovascular event.

## 2. Materials and Methods

### 2.1. Dataset Description

For this study, we utilized one hundred and thirty-four (134) asymptomatic cases with >50% carotid stenosis. The data were collected and anonymized from five (5) different clinical centers and were obtained under a data protection agreement fulfilling all the ethical and legal requirements for data sharing posed by the General Data Protection Regulation in a third-level care setting. These cases were examined using a 1.5-T whole-body system (Signa HDx, GE Healthcare, Waukesha, WI, USA) equipped with a bilateral four-channel phased-array carotid coil (Machnet BV, Eelde, The Netherlands). The subjects provided written informed consent to participate in the TAXINOMISIS study (www.clinicaltrials.gov; accessed on 15 May 2024; ID: NCT03495830) protocol, which received approval from the local ethics committee. The median age of the subjects was 69.4 years, and the vast majority suffered from hypertension (83%) and hypercholesterolemia (77.4%). Table 1 depicts the demographics data for the 134 subjects that were included in the present study.

Notably, 115 cases presented with no cerebrovascular events during the 3-year period, whereas 19 cases exhibited at least one cerebrovascular-related event such as stroke, transient ischemic attacks (TIA), myocardial infarctions (MI), or intracranial hemorrhages. As expected, the dataset was severely imbalanced due to the fact that the TAXINOMISIS study consisted of asymptomatic cases, which rarely trigger any events in a period of 3 years.

### 2.2. Methodology

#### 2.2.1. 3D Reconstruction

The process of reconstructing a 3D model of the carotid artery relies on a sequence of magnetic resonance images (MRI), involving ToF, T1w, T2w, and PD series. To briefly outline, the ToF sequence is employed to reconstruct the arterial lumen, while the fusion of T1w, T2w, and PD series facilitates the reconstruction of the arterial wall model and the model depicting plaque components. These three models are subsequently aligned in a later phase to generate the final arterial model. This reconstruction method is grounded in an innovative approach [30], which comprises three distinct steps:

Region of interest segmentation: The first step involves segmenting the regions of interest, including the lumen, outer wall, and plaque components. To accomplish this, three deep learning models were developed. Specifically, 485 tuples of ToF, T1w, T2w, and PD images from 42 different patients were annotated by two experts, resulting in a training dataset. This dataset was then utilized to train three UNET models, each tailored to segmenting a specific region of interest.

3D level set: Following segmentation, a morphological operator is applied to the 3D volume composed of stacked 2D segmented frames. This operation generates the 3D surface model representing the segmented regions.

3D meshing: Subsequently, the marching cubes algorithm is employed on the 3D surface model to produce the final reconstructed arterial model.

By employing this comprehensive approach, the reconstruction process yields detailed and accurate representations of the carotid artery, enabling further analysis and study of arterial pathologies.

#### 2.2.2. 3D Blood-Flow Simulations

The 3D reconstructed luminal carotid geometries underwent transient blood-flow simulations, utilizing patient-specific boundary conditions derived from the respective carotid UltraSound (US) screening. These conditions were comprised of flow velocity profiles obtained for a minimum of three consecutive cardiac cycles for each artery. In our simulations, blood flow was modeled using the Navier—Stokes and continuity equations, represented as Equations (1) and (2), respectively.

(1) $$\rho \frac{\partial v}{\partial t}+\rho (v•\nabla )v-\nabla •\tau =0,$$

(2) ∇•(ρv)=0.

Here, v denotes the blood velocity vector and τ stands for the stress tensor, defined by Equation (3) where *δij′* represents the Kronecker delta, *μ* is the blood dynamic viscosity, *p* indicates the blood pressure, and *εij* signifies the strain tensor as calculated by Equation (4).

(3) $$\tau =-p\delta_{ij}+2\mu \varepsilon_{ij},$$

(4) $$\varepsilon_{ij}=\frac{1}{2}\left(\nabla v+\nabla v^{T}\right).$$

The blood was assumed to follow Newtonian behavior with a density of 1050 kg/m^3^ and dynamic viscosity of 0.0035 Pa·s. All simulations were conducted using ANSYS^®^ v16.2 with a maximum element size set to 0.16 mm, consisting solely of tetrahedra. The mesh size was determined through a comprehensive sensitivity analysis, with a convergence criterion of 10^−4^ and an iteration limit of 150 for each time step.

In our effort to replicate patient-specific blood-flow dynamics accurately, we employ an approach that hinges on utilizing the derived mass flow rate profiles from Carotid Ultrasonography (US) images. These tailored profiles are applied to both the inlet, representing the proximal boundary of the Common Carotid Artery (CCA), and the outlet of the External Carotid Artery (ECA). This methodological choice is substantiated by the patient-specific US data, which provide detailed flow velocity profiles for at least two of the three arterial branches: CCA, Internal Carotid Artery (ICA), and/or ECA.

Each US image serves as a visual repository, providing insights into intricate velocity measurements and corresponding waveforms for multiple cardiac cycles across various arteries. The patient-specific flow velocity diagram is created using only the peak systolic velocity (PSV) and the end diastolic velocity (EDV) values using a dedicated in-house developed MATLAB script.

Additionally, patient-specific cardiac cycle durations are computed based on pulse rate information, incorporating temporal dynamics into the simulations. This comprehensive methodology not only leverages the richness of the US data but also precisely integrates this information, ensuring the utmost accuracy in capturing the nuances of patient-specific blood-flow dynamics. The velocity diagram is then transformed into a mass flow rate diagram, using Equation (5).

(5) $$\dot{m}=\rho VA,$$

**Figure 1:**
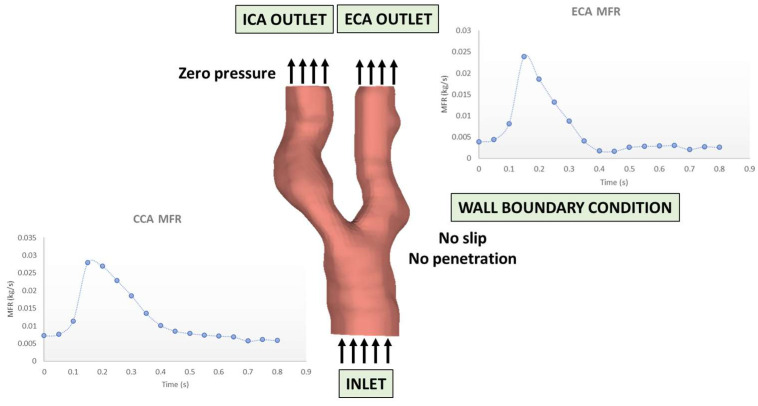
Boundary conditions for an indicative case.

**Figure 2:**
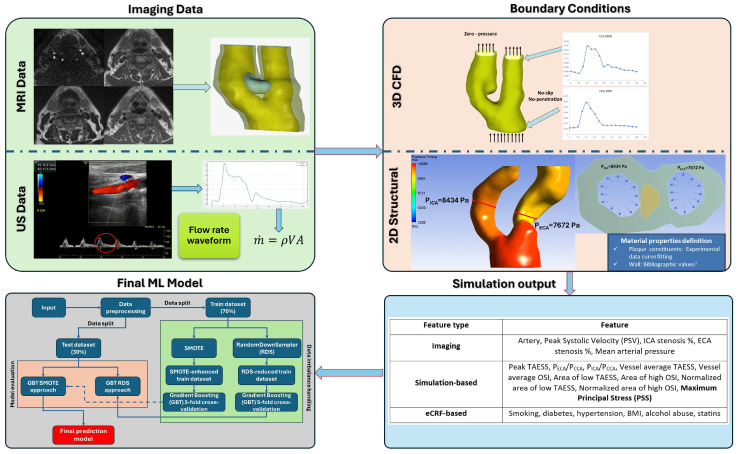
Overall pipeline of the proposed approach. The red circle in green box within the US image indicated the cardiac cycle from which the diagram next to it derives. The literature values indicated in the blue box with ^1^ are derived from [32].

The symbol $\dot{m}$ represents the mass flow rate, ρ denotes the blood density, V signifies the blood velocity, and A represents the cross-sectional area at the velocity measurement location. Regarding the internal carotid artery (ICA), a zero-pressure boundary condition is applied, with a detailed explanation provided in the results section for the rationale behind this choice. Additionally, a no-slip boundary condition is enforced at the arterial wall boundary, ensuring zero velocity at the wall, while a no-penetration condition is imposed, preventing fluid from passing through the boundary. Figure 1 depicts the boundary conditions for an indicative case of the utilized dataset. Briefly, the entire modeling process consists of the following steps: (a) 3D reconstruction of the lumen, the arterial wall and the plaque constituents deriving from the MRI data and 2D cross-section reconstruction for the site of maximum plaque burden; (b) flow velocity profiles extraction for the CCA and ECA deriving from the US data and transformation to the respective mass flow rate profiles for both, respectively; (c) 3D CFD blood-flow simulation using the 3D model of the lumen, accompanied by the mass flow rate profiles for the CCA and ECA and an a zero-pressure value for the ICA, serving as boundary conditions for the FEM simulation; (d) 2D structural analysis, using the 2D model for the site of maximum plaque burden and the maximum pressure at this specific site, as calculated by (c); (e) calculation of the following hemodynamic parameters: Peak TAESS, P_ECA_/P_CCA_, P_ICA_/P_CCA_, vessel average TAESS, vessel average OSI, normalized area of low TAESS, Normalized area of high OSI, plaque structural stress (PSS); (f) input of the aforementioned parameters in the proposed ML model; (g) event risk score calculation. The brief description of the entire pipeline is depicted in Figure 2.

#### 2.2.3. Structural Analysis

**Figure 3:**
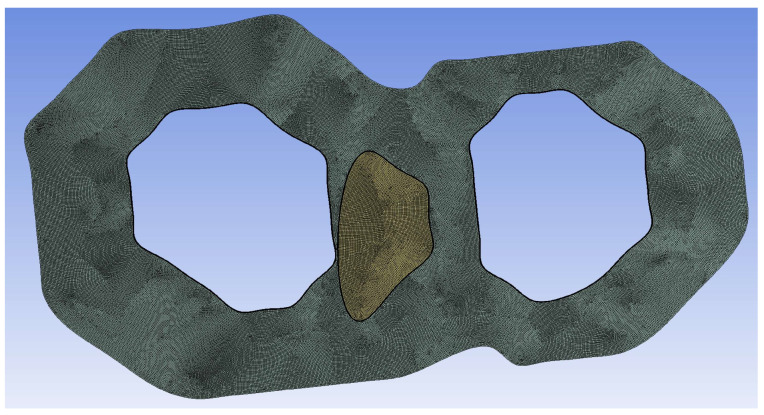
2D mesh for the arterial model with the plaque component indicated in yellow. An inflation process was followed in the two luminal borders, as well as at the borders of the fibrous plaque.

After having performed the 3D blood-flow simulation in order to calculate the hemodynamic parameters to be fed into the machine learning model, as well as the pressure distribution throughout the entire cardiac cycle, the next step is to perform a 2D structural analysis of the cross-section which exhibits the highest plaque burden value that has already been calculated by the 3D reconstruction module. The 2D pixel coordinates of the lumen, the arterial wall, and the plaque components are transformed to cartesian coordinates and the final 2D geometry is then discretized into quadrilateral elements with a maximum face size of 0.05 mm. furthermore, an inflation process is performed at the interface between the plaque component and the arterial wall, as well as at the borders of the arterial wall and the lumen (Figure 3). Regarding the utilized boundary conditions for the 2D-analysis, the outer perimeter of the external wall is assumed to act as a frictionless support, aiming to prevent any potential displacement of the adventitia. Simultaneously, the plaque components are bonded with the arterial wall throughout the simulation. The luminal boundary emerges as a crucial interface, serving as the platform onto which the previously computed maximum site-specific pressure, derived from the aforementioned 3D blood-flow simulation, is applied as a loading condition. The pressure values obtained at the cross-section, which aligns with the segmented slice, are crucial for guiding the further stages of analysis. This thorough approach ensures the model’s integrity and allows for a detailed understanding of the real-world complexities involved in simulating the biomechanical behavior of arterial structures.

**Figure 4:**
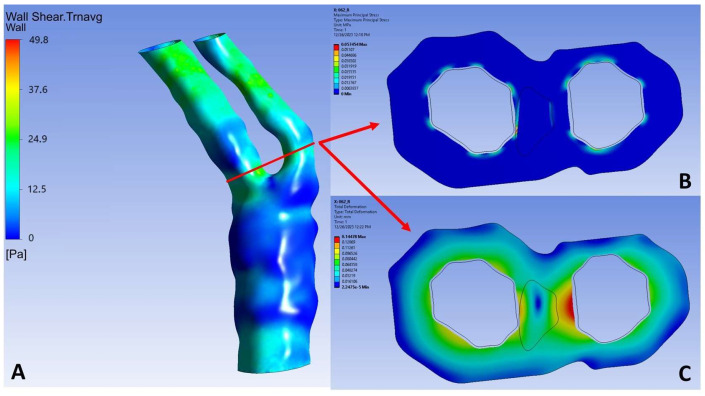
PSS and deformation calculations for a representative case (right carotid). (**A**) The initial 3D blood-flow simulation is used to depict the exact location at which the structural analysis was performed. The location exhibited the highest plaque burden value in the entire arterial length. (**B**) PSS calculated at 57.4 kPa, at the ICA, between the fibrous plaque and the arterial lumen. (**C**) Maximum deformation calculated at the inner border of the ECA (0.14 mm).

Following this, the structural analysis progresses by assigning specific material properties to the arterial wall and the plaque being examined within the selected cross-section. For both the arterial wall and the fibrous plaque, hyperelastic material properties are assigned. Regarding the arterial wall, we employed the Mooney–Rivlin approach [31], defined by the parameters C_10_ = 0.07 MPa, C_20_ = 3.2 MPa, C_21_ = 0.0716 MPa, while setting the remaining parameters as C_ij_ = 0 systematically. To address incompressibility, the parameter d of the arterial wall was set at d = 1.0 × 10^−5^ Pa^−1^. The material properties attributed to the plaque components were determined through the analysis of the stretch ratio to Cauchy stress diagrams which were created experimentally [32]. These diagrams formed the basis for calculating and extracting the essential 5-parameter Mooney–Rivlin constants. The resulting values, crucial for accurately characterizing the mechanical behavior of the plaque components, are shown in Table 2. Figure 4 depicts the pipeline for the calculation of PSS values, beginning with the calculation of the maximum pressure during the cardiac cycle at the site of the cross-section of interest, followed by the 2D structural simulation using the aforementioned calculated pressure as a loading boundary condition.

#### 2.2.4. Hemodynamic Features Used as Input for the ML Model

A total of 134 cases were analyzed to compute the necessary hemodynamic parameters for the event prediction model. This analysis included fluid-related hemodynamic parameters, as well as structural-derived parameters. Specific emphasis is given to deriving the highest plaque structural stress (PSS) value from each case via 2D structural analysis [33,34]. This value, acting as a biomechanical marker of vulnerability, is crucial for our predictive modeling, especially for the plaque rupture model. Apart from the PSS values, the 3D simulation produces metrics such as the Time-Averaged Wall Shear Stress (*TAWSS*) (Equation (6)), the average Oscillatory Shear Index (*OSI*) values (Equation (7)) and distributions throughout the artery, the pressure drop between the inlet (CCA boundary), and the two outlets (ICA and ECA boundaries), respectively:

(6) $$TAWSS=\frac{1}{T}\int_{0}^{T}\left|\tau_{w}\right|dt,$$

(7) $$OSI=\frac{1}{2}\left[1-\frac{\left|\frac{1}{T}\int_{0}^{T}\tau_{w}dt\right|}{\frac{1}{T}\int_{0}^{T}\left|\tau_{w}\right|dt}\right].$$

where *T* is a full cardiac cycle and *τ_w_* is the instantaneous WSS. The OSI can be considered as the fraction of the angle and the magnitude change between the instantaneous wall shear stress and the time-averaged wall shear stress. The values that can be assigned to it range from 0–0.5. While 0.5 is a value that represents an unstable, oscillatory flow with a WSS of 0 Pa, 0 denotes a wholly unidirectional WSS. Areas with high OSI values are typically more likely to form plaque and exhibit endothelial dysfunction [35,36]. Atherogenesis has also been shown to be more likely to manifest at very low TAWSS values (0.4 Pa), whereas high TAWSS values (>40 Pa) may lead to endothelial dysfunction and subsequent damage, raising the likelihood of thrombosis [37,38].

#### 2.2.5. Problem Definition

**Figure 5:**
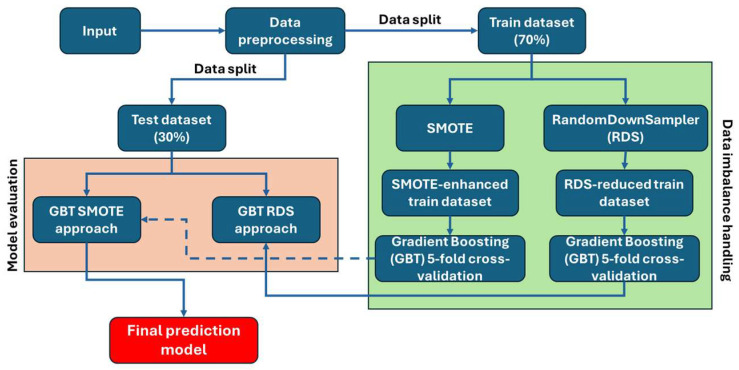
ML model development workflow.

The development of the final model for predicting cerebrovascular events is framed as an advanced multivariate binary classification challenge, focusing on data from the electronic Case Report Form (eCRF) repository of our dataset. This classification delineates between class 0 (cases without events) and class 1 (cases with cerebrovascular events). Figure 5 depicts the ML model development pipeline which was followed. Given the imbalanced nature of the dataset (115 cases with no events and 19 cases with cerebrovascular events), with a significantly lower number of positive instances compared to negative instances, we applied several preprocessing steps to ensure robust model training and evaluation.

Table 3 shows the features used by the machine learning (ML) event prediction model which combines imaging information, both 2D and 3D simulation-based features, as well as eCRF-based data.

Data Encoding

Categorical features in the dataset were encoded using one-hot encoding to transform them into a numerical format suitable for machine learning algorithms. This step ensures that the model can process categorical data efficiently.

Data Splitting

The dataset was split into training and testing subsets using a 70–30 split. This division was performed using stratified sampling to maintain the same proportion of positive and negative instances in both the training and testing sets, preserving the original class distribution. The training dataset included 80 class 0 instances and 13 class 1 instances, whereas the test dataset included the remaining 35 class 0 instances and the remaining six class 1 instances, respectively.

Dataset-imbalance-handling approaches

Two imbalance-handling approaches were implemented, (a) SMOTE Oversampling for Class 1 and (b) Downsampling for Class 0, respectively.

- SMOTE Oversampling (Class 1)

The Synthetic Minority Oversampling Technique (SMOTE) [39] was employed to address class imbalance by generating synthetic samples for the minority class. This technique interpolates between existing minority class samples to create new instances, thereby balancing the class distribution without reducing the number of majority class instances. SMOTE was applied to the training set, generating synthetic samples to balance the classes. After applying SMOTE (Equation (8)), the values in the specified binary columns were rounded and clipped to ensure they remained binary (0 or 1):

(8) $$SMOTE-generated=\frac{\sum \left(Minority\;sample\;pairs\right)}{Number\;of\;pairs}.$$

b. Downsampling Class 0 (Majority class)

Random downsampling without replacement was applied based on the “RandomUnderSampler” approach [40] to address class imbalance by reducing the number of majority class instances to equally match the number of minority class instances. This approach prevents the model from being biased towards the majority class by preserving the same number of instances in both classes. The “RandomUnderSampler” was applied to the training dataset to yield equally balanced populations as in:

(9) $$Downsampling=\frac{\sum \left(Randomly\;selected\;majority\;samples\right)}{Number\;of\;majority\;samples}.$$

Model Training and Cross-Validation

The Gradient Boosting Tree (GBT) classifier was chosen to solve a binary classificaiton problem (“0”: asbence of events, “1”: presence of events) based on its effectiveness in handling complex, non-linear relationships and robustness to overfitting. We performed 5-fold stratified cross-validation on the training set to evaluate the model’s performance in both approaches (SMOTE approach and downsampling approach). The Gradient Boosting Tree (GBT) classifier is a powerful ensemble learning algorithm which has been designed to optimize predictive performance by combining multiple weak learners, typically decision trees, into a strong learner. It involves the construction of a sequence of models, where each model focuses on correcting the errors of its predecessor. The models are finally combined to produce the predictive model. The learning process is briefly described below.

A base model $F_{0}\left(x\right)$ is initially defined as follows:

(10) $$F_{0}\left(x\right)=\arg\underset{c}{\min}\sum_{i=1}^{n}L\left(y_{i},c\right),$$

where *L* is the loss function, $y_{i}$ are the true values, and *c* is a constant. An iterative boosting is then applied for *m* = 1, 2…, *M* stages and the pseudo-residuals, say $r_{im}$, are estimated as in:

(11) $$r_{im}=-{\left[\frac{\partial L(y_{i},F(x_{i}))}{\partial F(x_{i})}\right]}_{F=F_{m-1}},$$

where $\partial L(y_{i},F(x_{i}))$ is the gradient of the loss function with respect to the predictions, and $F_{m-1}$ is the model from the *m*−1 stage. A weak learner is then applied to the pseudo-residuals, yielding:

(12) $$h_{m}(x)=\arg\underset{h}{\min}\sum_{i=1}^{n}{\left(r_{im}-h(x_{i})\right)}^{2},$$

where $h_{m}(x)$ is the weak learn at stage m and ${\left(r_{im}-h(x_{i})\right)}^{2}$ is the squared error between the pseudo-residuals and the weak learner’s predictions. The model at stage *m*, say $F_{m}\left(x\right)$, is then incrementally updated through the following rule:

(13) $$F_{m}\left(x\right)=F_{m-1}\left(x\right)+vh_{m}(x),$$

where v is the learning rate which reflects the contribution of each weak learner. The final model (i.e., the model after *M* stages, $F_{M}\left(x\right)$) is defined as the sum of the base model with the contributions from all the weak learners:

(14) $$F_{M}\left(x\right)=F_{0}\left(x\right)+\sum_{m=1}^{M}vh_{m}(x).$$

We employed everything in Python 3.11 using the “GradientBoostingClassifier” from the scikit-learn package, where the number of estimators (i.e., number of boosting stages) was set to 100, the learning rate to 0.1, the loss was set to the logarithmic loss, and the max. depth of the individual regression estimators to 3. The quality of each split was measured using the mean squared error with improvement score by Friedman.

## 3. Results

### 3.1. Internal Validation of Both Approaches on the Training Dataset

The utilized model performance metrics for the comparison of the two imbalance handling approaches are the balanced accuracy, the negative predictive value, the positive predictive value, the area under the receiver operating curve (ROC AUC), and the sensitivity and specificity. The values of the adopted performance metrics and their mean value and the 5-fold standard deviation are given in Table 4 for the SMOTE approach and in Table 5 for the downsampling approach.

The results of the 5-fold cross-validation scheme revealed a slight superiority of the SMOTE approach over the downsampling approach, something that was somehow expected due the nature of the SMOTE process, which increases the minority class instances to match the instances of the majority class regarding the training dataset. The downsampling approach, on the other hand, produced relatively satisfactory internal validation results, even though the minority class instances within the training data were very few. Given the relatively small number of positive instances in our dataset, using five folds ensures that each fold contains a sufficient number of samples from both classes, maintaining the class distribution and providing more stable and reliable performance estimates compared to higher fold values like 10-fold, which might result in folds with very few positive instances. This approach also mitigates the risk of overfitting and ensures that the performance metrics we obtain are more reflective of the model’s ability to generalize to unseen data, making it a robust choice for validating our predictive models.

**Figure 6:**
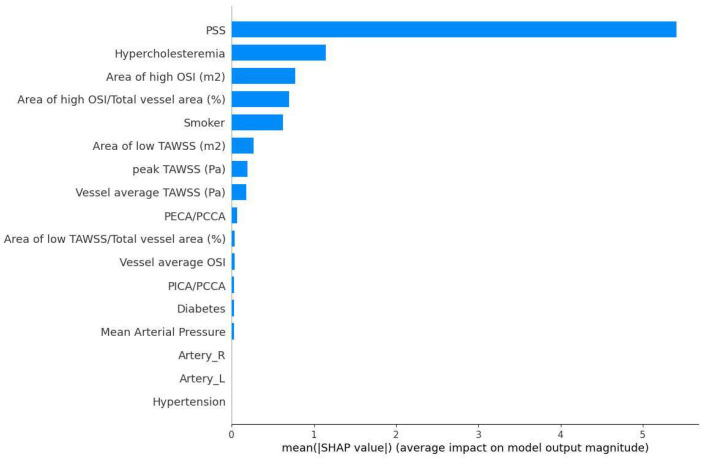
Feature importance based on mean SHAP values in the SMOTE approach. PSS and Vessel average TAWSS values are indicated as the most significant features.

SHapley Additive exPlanations (SHAP) analysis was performed to explain the predictions made by the proposed model by calculating the influence of each feature to the overall event prediction [41]. The most influential features of the proposed ML model using the SMOTE approach are presented in Figure 6. The mean calculated SHAP values for all the utilized features were calculated to reflect the global feature importance.

These features represent the most significant predictors of the event outcome in the dataset, with the PSS (plaque structural stress) being the most influential, followed by the presence of hypercholesterolemia and the areas of high OSI.

### 3.2. Testing of Both Approaches on the Test Dataset

The performance of the SMOTE approach on the test dataset demonstrated its effectiveness in addressing class imbalance and improving the model’s ability to predict cerebrovascular events. The SMOTE-enhanced model achieved a classification outcome that indicated balanced sensitivity and specificity, ensuring that both positive and negative instances were accurately identified. Specifically, the confusion matrix for the SMOTE approach revealed 32 true negatives, three false positives, two false negative, and four true positives. This translated to a high specificity rate, indicating a strong ability to correctly identify non-events, while maintaining a reasonable sensitivity rate for detecting actual events. The ROC AUC score further supported the robustness of this model, showcasing its inherent ability in distinguishing between the positive and negative classes.

**Figure 7:**
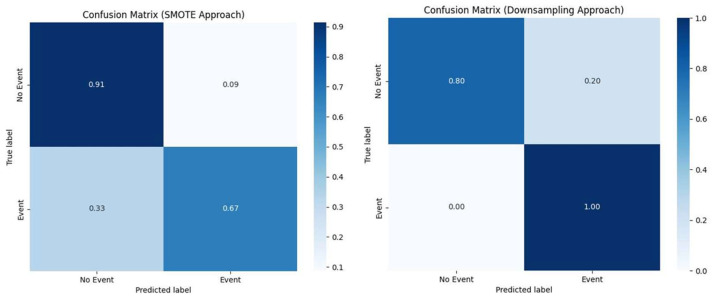
Normalized **c**onfusion matrices for the SMOTE approach (**left image**) and Downsampling approach (**right image**), respectively.

In contrast, the “RandomUnderSampler” approach, also exhibited promising results on the test dataset. The confusion matrix for this method showed 28 true negatives, seven false positives, zero false negatives, and six true positives. Figure 7 depicts the normalized confusion matrices for the two approaches. The ROC AUC score was also competitive, reflecting its balanced performance across both classes. Overall, both approaches demonstrated their respective strengths, with the SMOTE approach presenting slightly superior results in all metrics.

The performance of the GBT classifier for both class imbalance approaches is depicted in Table 6.

## 4. Discussion

In this work, we developed a robust predictive model for cerebrovascular events by integrating finite element-derived simulation results with imaging and non-imaging clinical data. Our methodology involved comprehensive data preprocessing, including the encoding of categorical variables. To address the significant class imbalance in our dataset (115 class 0 vs. 19 class 1 instances), we employed two distinct techniques: “RandomUnderSampler” and Synthetic Minority Oversampling Technique (SMOTE). The “RandomUnderSampler” method undersamples (or downsamples) the majority class to create a more balanced training set, while SMOTE generates synthetic samples for the minority class, enhancing the model’s ability to learn from both classes. We then trained a GBT classifier, leveraging its strength in handling complex, non-linear relationships within the data.

The performance of our predictive model was initially evaluated on the transformed train datasets using a 5-fold cross-validation strategy to ensure reliable and generalizable results. This strategy maintained the class distribution within each fold, providing robust performance estimates. The results indicated that both the “RandomUnderSampler” and SMOTE approaches effectively improved the model’s sensitivity and specificity. As indicated in the generated results, the SMOTE approach was the selection of preference, since it produced a more balanced performance, compared to the “RandomUnderSampler” approach. Both methods achieved balanced accuracy and high ROC AUC scores, confirming the model’s capability to distinguish between positive and negative instances. These results underscore the effectiveness of our approach in creating a reliable predictive model for cerebrovascular events. The final GBT model (SMOTE-enabled) exhibited a balanced accuracy of 88% along with an AUC of 0.91. The performance of our approach compared to other ML-based models from literature is presented in Table 7.

The integration of finite element-derived simulation results as input features represents a significant innovation in our predictive modeling framework. By combining these sophisticated simulation outputs with imaging and non-imaging data, we have enriched the feature space, enabling the model to capture more nuanced patterns associated with cerebrovascular events. This multidisciplinary approach leverages the strengths of advanced computational techniques and clinical insights, providing a more comprehensive understanding of the factors contributing to cerebrovascular risks. The finite element simulations offer detailed biomechanical insights that are not typically available from clinical data alone, thus enhancing the model’s predictive power.

Our method’s innovative aspect lies in this unique combination of data types, which has not been extensively explored in previous studies. The inclusion of simulation-derived features allows for a more detailed characterization of the vascular environment, potentially leading to earlier and more accurate predictions of cerebrovascular events. This approach not only demonstrates the feasibility of integrating diverse data sources but also sets a precedent for future studies aiming to leverage advanced simulations in clinical predictive models. By incorporating these elements, our work provides a novel framework that could be adapted and expanded for other clinical applications, ultimately contributing to more personalized and precise healthcare interventions.

However, the main limitation of our work lies in the rather small dataset that was available. The dataset was relatively imbalanced, a fact that can obviously be attributed to the fact that cerebrovascular events are less than common in asymptomatic patients with carotid artery disease. This was partially tackled by using a 5-fold cross-validation technique, as well as by trying an oversampling (SMOTE) technique for the minority class and calculating the results of the newly acquired enhanced dataset, as well as by applying a downsampling approach for the majority class to match the size of the minority class and recalculating the newly acquired dataset. Obviously, in the presence of a higher number of recorded events which would lead to a more balanced dataset, a different validation strategy would have been followed, having an obviously larger test subset. Furthermore, the proposed method relies on the accuracy of the 3D reconstruction of the carotid vasculature, as well as on the accuracy of the plaque characterization process performed. Both aspects are highly dependent on the quality of the acquired MRI images, a fact that can easily affect the calculated hemodynamic parameters which, in turn, affect the risk of cerebrovascular events that is calculated through the ML model.

The clinical impact of our work is of great importance, since it covers a sensitive subset of carotid artery disease patients who are asymptomatic but also have carotid artery stenoses of >50%. Our model addresses a critical gap in current clinical practice by providing a risk stratification beyond the conventional stenosis threshold of 80% used for surgical interventions. Specifically, it can identify patients with stenoses less than 80% who are at high risk of cerebrovascular events, thereby enabling earlier intervention and potentially preventing strokes that might otherwise be missed. Conversely, the model can also discern patients with stenoses greater than 80% who are at low risk, thus preventing unnecessary surgical procedures and their associated risks. By refining the decision-making process for intervention, our model has the potential to improve patient outcomes, reduce healthcare costs, and contribute to more personalized and effective management of carotid artery disease.

Our future work will focus on acquiring significantly larger datasets to enhance the robustness and generalizability of our predictive model for cerebrovascular events. These expanded datasets will aim to include a diverse range of cases, particularly those involving lipid-rich or calcified plaques, which are critical factors in the development and progression of cerebrovascular disease. By incorporating these additional cases, we can further refine our model to capture the full spectrum of clinical scenarios, thereby improving its predictive accuracy and reliability. Moreover, larger datasets will allow for more sophisticated machine learning techniques, such as deep learning, to be explored and potentially implemented. These techniques can leverage the increased data volume to identify even more subtle and complex patterns, ultimately leading to more precise and personalized predictions. Additionally, future studies will investigate the integration of other emerging data sources, such as genetic and molecular profiles, to further enhance the predictive power and clinical applicability of our model.

## 5. Conclusions

Our main goal was to develop a predictive ML model for identifying the likelihood of future cerebrovascular incidents, with a focus on asymptomatic cases. To accomplish this, we utilized a dataset that included standard non-imaging information from electronic Case Report Forms (eCRFs), imaging data from ultrasound (US) and magnetic resonance imaging (MRI), hemodynamic and structural parameters obtained from 2D and 3D finite element method (FEM) simulations. Despite the challenge of working with an imbalanced dataset, a natural outcome due to the rarity of events among asymptomatic patients, our methodology produced encouraging outcomes. The peculiarities of asymptomatic cases, mainly the reduced frequency of varied plaque compositions like lipid-rich plaques or hemorrhages, presented a constraint in data diversity. However, our model showcased notable accuracy and specificity in forecasting cerebrovascular events, affirming its value for clinical application.

Our workflow, which includes 3D reconstruction, blood-flow simulation, 2D structural analysis, and risk assessment, was proven to be efficient. This streamlined approach requires limited computational efforts and time, completing in just 15–20 min on average, although this may vary with the quality of imaging and the artery length examined. This efficiency, combined with the model’s accuracy, underscores our method’s practicality and effectiveness.

## Figures and Tables

**Table 1:** Patient demographics info.

Patients (*n* = 134)	N (%)
Age (years)	69.4 ± 8.4
Gender (male)	87 (65.1)
**Risk factors**	
Smoking	94 (70.1)
Diabetes mellitus	47 (35)
Hypertension	111 (83)
Hypercholesterolemia	104 (78)
Coronary disease	35 (26.4)
Obesity	18 (13.5)
BMI	26.48 ± 3.4

**Table 2:** Mooney–Rivlin parameters for the fibrotic plaque components as derived from [32].

Material	C_10_ (Pa)	C_01_ (Pa)	C_20_ (Pa)	C_11_ (Pa)	C_02_ (Pa)	d
**Fibrosis**	−3.3232 × 10^6^	3.4296 × 10^6^	4.5387 × 10^8^	−1.021 × 10^9^	5.8132 × 10^8^	1 × 10^−5^

**Table 3:** Utilized features for the event prediction model.

Feature type	Feature
**Imaging**	Artery, peak systolic velocity (PSV), ICA stenosis %, ECA stenosis %, mean arterial pressure
**Simulation-based**	Peak TAESS, P_ECA_/P_CCA_, P_ICA_/P_CCA_, vessel average TAESS, vessel average OSI, normalized area of low TAESS, Normalized area of high OSI, plaque structural stress (PSS)
**eCRF-based**	Smoking, diabetes, hypertension, BMI, alcohol abuse, statins

**Table 4:** Evaluation of the cerebrovascular event prediction problem over 5-fold using both imaging- and non-imaging-derived data on the training dataset using the SMOTE approach.

Fold	Balanced Accuracy	NPV	PPV	AUC	Sensitivity	Specificity
0	0.91	0.88	0.93	0.99	0.88	0.94
1	0.97	1	0.94	1	1	0.94
2	1	1	1	1	1	1
3	0.84	0.88	0.82	0.84	0.88	0.81
4	0.94	1	0.89	1	1	0.88
**Mean**	0.93	0.95	0.92	0.96	0.95	0.91

**Table 5:** Evaluation of the cerebrovascular event prediction problem over 5-fold using both imaging- and non-imaging-derived data on the training dataset using the downsampling approach.

Fold	Balanced Accuracy	NPV	PPV	AUC	Sensitivity	Specificity
0	1	1	1	1	1	1
1	0.75	0.50	1	0.75	0.50	1
2	0.67	1	0.5	0.67	1	0.33
3	1	1	1	1	1	1
4	0.83	0.67	1	1	0.67	1
**Mean**	0.85	0.83	0.9	0.88	0.83	0.87

**Table 6:** Aggregated performance results for both approaches on the test dataset.

Class Imbalance Approach	Accuracy	ROC AUC	Recall	Specificity	F1-Score
**SMOTE**	88%	0.92	0.88	91%	0.88
**Downsampling**	83%	0.91	0.83	80%	0.85

**Table 7:** Comparison of our ML-based approach to current literature on ML-based predictive models for cerebrovascular-related events.

Publication	Accuracy	Specificity	AUC	F1-Score
Wu, X., et al. [25]	0.85	0.82	0.87	0.84
Weng, S., [26]	0.88	0.86	0.9	0.87
Xia, H., [27]	0.83	0.8	0.84	0.82
Yun, K., [28]	0.9	0.88	0.91	0.89
Bin, C., [29]	0.84	0.81	0.85	0.83
**Our approach**	0.88	0.91	0.92	0.88

## Data Availability

The data used in this study can be available upon request from the corresponding author.
